# A Brief, Digital Music-Based Mindfulness Intervention for Black Americans With Elevated Race-Based Anxiety and Little-to-No Meditation Experience (“healing attempt"): Replication and Extension Study

**DOI:** 10.2196/53268

**Published:** 2023-11-24

**Authors:** Grant Jones, Franchesca Castro-Ramirez, Maha Al-Suwaidi, Taylor McGuire, Felipe Herrmann

**Affiliations:** 1 Department of Psychology Harvard University Cambridge, MA United States; 2 Department of Psychology University of California Los Angeles, CA United States

**Keywords:** Black music, mindfulness, meditation, music, song, psychotherapy, self-compassion, ethnic, cultural, single-case experiment, race, anxiety, digital health intervention, Black, digital health, low income, racial disparity, mental health

## Abstract

**Background:**

Race-based anxiety is a critical health issue within the Black community. Mindfulness interventions hold promise for treating race-based anxiety in Black Americans; however, there are many barriers that prevent Black Americans from using these treatments, such as low cultural relevance, significant time burdens, and excessive costs.

**Objective:**

This study is a replication and extension of findings that “healing attempt”—a brief (<60-minute), digital, music-based mindfulness intervention—is a feasible and acceptable intervention for race-based anxiety in Black Americans. In this study, we tested this research question among those with little-to-no meditation experience.

**Methods:**

The participants were 4 Black American adults with elevated race-based trait anxiety and little-to-no meditation experience. We used a series of multiple-baseline single-case experiments and conducted study visits on Zoom (Zoom Video Communications) to assess whether the intervention can decrease state anxiety and increase mindfulness and self-compassion in Black Americans. We also assessed feasibility and acceptability using quantitative and qualitative scales.

**Results:**

In line with our hypotheses, “healing attempt” increased mindfulness/self-compassion (Tau-U range: 0.57-0.86; *P*<.001) and decreased state anxiety (Tau-U range: –0.93 to –0.66; *P*<.001), with high feasibility and acceptability (the average likelihood of recommending “healing attempt” was 88 out of 100).

**Conclusions:**

“healing attempt” may represent a feasible intervention for race-based anxiety in Black Americans with elevated race-based anxiety and little or no mindfulness experience. Future between-subjects randomized feasibility trials can assess whether the intervention can give rise to lasting improvements in race-based anxiety, mindfulness, and self-compassion.

**Trial Registration:**

OSF Registries osf.io/k5m93; https://osf.io/k5m93

## Introduction

Black Americans experience heightened anxiety due to racial discrimination (ie, race-based anxiety) [1-3]. Furthermore, there are pervasive disparities in access to evidence-based mental health interventions and anxiety treatments that prevent Black Americans from getting care for race-based anxiety [4,5]. Although the Black community is afflicted with increased anxiety due to race-based discrimination, there are a limited number of studies that have investigated avenues for treating race-based anxiety in Black Americans [6-10]. Additional research is needed to produce interventions for Black Americans that target anxiety that stems from racism and discrimination.

Mindfulness is a Buddhist concept that entails paying attention to the present moment in a nonjudgmental and accepting way [11], and interventions that cultivate mindfulness have the potential to alleviate race-based anxiety in the Black community. Systematic reviews and meta-analytic evidence have demonstrated the efficacy of mindfulness-based interventions for decreasing anxiety symptoms [12-14], and cross-sectional studies have shown that trait mindfulness moderates the severity of anxiety and depression symptoms stemming from racism and discrimination in the Black community [15-17]. Hence, mindfulness interventions hold promise in decreasing race-based anxiety among Black Americans.

Regardless of the potential of mindfulness-based interventions, three central obstacles impede the Black community from accessing and using these resources. First, most mindfulness interventions are not culturally adapted to resonate with Black Americans, despite meta-analytic consensus indicating the superior effectiveness of culturally adapted interventions compared to nonadapted interventions [18-20]. Second, financial barriers may prevent access to mindfulness-based psychological treatment among Black Americans. For instance, mindfulness-based stress reduction (MBSR)—the gold standard of mindfulness interventions—regularly costs ~US $1300 [21]. Given that the racial wealth gap in the United States has persisted for over 50 years and a Black family’s median income has remained two-thirds the size of a White family’s income [22-24], steep costs can prevent access to wellness resources for Black Americans. Third, mindfulness interventions often take up large amounts of time (eg, MBSR is traditionally an 8-week course lasting 26 hours) that may pose barriers to engagement in the Black community. Several qualitative studies have indicated that busy home schedules, excessive traveling demands, and difficulties carving out time can discourage Black Americans from using mindfulness resources and engaging in formal meditation practice [25-28]. Additional research is needed toward more practical ways of embedding mindfulness within the Black community.

Therefore, GJ created “healing attempt”—a brief (<60 min), digital music–based intervention—to address the aforementioned barriers. This 25-minute intervention consists of prerecorded and originally composed poems, guided meditations, and songs that are meant to cultivate mindfulness for Black Americans. The intervention includes background music tracks that stem from Black diasporic music traditions (eg, soul, gospel, and jazz). The intervention also includes sounds from nature, as this principle is often discussed in the music therapy literature as aiding with anxiety reduction for music-based interventions [29].

Our team previously conducted 2 studies examining the feasibility of “healing attempt” for treating race-based anxiety in Black Americans [30,31]. Both prior studies used a multiple baseline design consisting of repeatedly assessing state anxiety (ie, momentary anxiety) and state mindfulness/self-compassion and comparing changes in these metrics across two time-periods: (1) an initial baseline period with no intervention (ie, the “control” period) and (2) an experimental period where the intervention is administered [32]. The initial study (N=5) recruited middle-to-low income Black Americans (<US $50,000/year) experiencing elevated race-based anxiety, and the study demonstrated that “healing attempt” alleviated anxiety with high feasibility and acceptability (eg, the average likelihood of recommending the intervention was 98 out of 100) [30]; the second study (N=8) replicated these findings (ie, decrease in state anxiety and high feasibility scores) and additionally found that the intervention simultaneously increased mindfulness/self-compassion [31].

Despite these encouraging findings, there is a need to replicate and further extend our previous work. In both of our prior studies, most participants had familiarity with meditation, as we aimed for the initial pilot studies to consist of individuals with prior meditation experience who can make an informed evaluation of whether “healing attempt” is a suitable vehicle for mindfulness for the Black community. In this multiple-baseline experiment, we recruited Black American participants (N=4) with little-to-no familiarity with meditation to assess whether the intervention was feasible for individuals less acquainted with mindfulness. In light of the replication crisis in psychology, we decided a priori to conduct this additional, stand-alone replication study to attempt to incrementally build a solid base of evidence for this intervention. Such a study can provide preliminary evidence that “healing attempt” may be acceptable for Black Americans who rarely meditate or have never meditated before, potentially expanding access to mindfulness-based resources beyond those who currently have access and interest.

## Methods

### Overview

The participants were 4 Black American adults with elevated race-based anxiety. We recruited participants from Prolific, a web-based study recruitment platform that we have used in both prior studies of this intervention.

**Figure 1:**
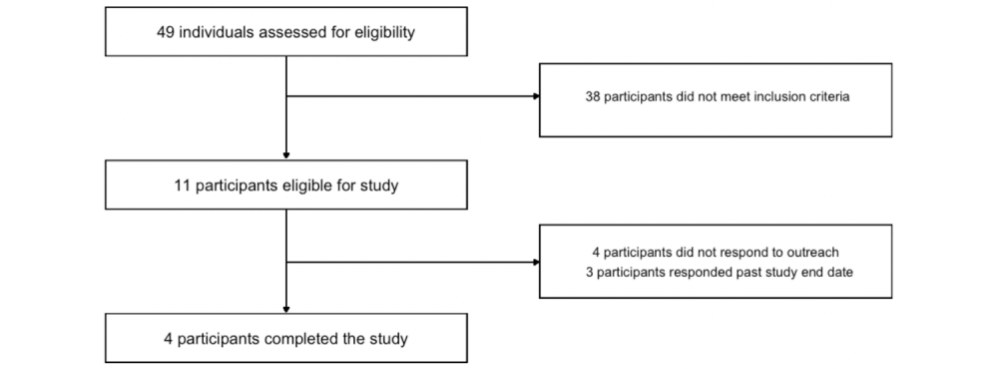
CONSORT diagram of participants. CONSORT: Consolidated Standards of Reporting Trials.

First, we used the screening tool included in the Prolific platform to select Black American adults who endorsed elevated anxiety, were middle-to-low income (<US $50,000/year), and reported not engaging in meditation. We screened 49 of these potential participants to select individuals with elevated race-based trait anxiety (State-Trait Anxiety Inventory-Trait-5 [STAIT-5] scores ≥12 + ≥60 on a scale of 0 [not at all] to 100 [entirely] for the following question; “If you are a racial or ethnic minority, how much do you feel that racism and/or discrimination contribute to your elevated anxiety levels?”). With our study-specific screener, we also confirmed that potential participants had little-to-no familiarity with meditation (score of ≤2 on a scale of 1 [no meditation experience] to 5 [meditation is central in my life] for the following question: “How familiar are you with meditation and/or mindfulness?”). Participants were paid US $1 for completing the screener. Overall, 11 individuals were eligible for our study and we ultimately scheduled and completed study visits with 4 participants. Figure 1 presents a diagram of the flow of participants through our study.

All study visits were conducted on Zoom, and participants were required to have their video cameras on during the study visit. This format allowed us to directly observe and confirm participants’ engagement with the intervention. The study consisted of 2 main study phases—the “A” (baseline) phase and the “B” (experimental) phase—during which we assessed our main outcomes (state anxiety and mindfulness/self-compassion) in 2-minute intervals. For the “A” phase, participants were randomized and assigned to 1 of 4 baseline conditions of differing lengths (10, 12, 14, or 16 minutes of baseline; 5, 6, 7, or 8 assessment periods, respectively). In this phase, participants were free to engage in any activities they wished (ie, sitting in silence and reading a book). Next, during the “B” phase, we administered “healing attempt;” as the intervention is 25 minutes long, there were 12 measurement periods in the “B” phase. The only instruction for this phase of the study was for participants to assume a comfortable body position and to attend to any instructions they received within the intervention. Following the intervention, we administered study-specific quantitative and qualitative questions to assess feasibility and acceptability. Participants were paid US $60 for completing a study visit.

### Ethical Considerations

This study protocol was approved by the Harvard University IRB (IRB21-0256).

### Measures

#### State Anxiety

We used the State Trait Anxiety Inventory-6 (STAI-6) to assess state anxiety in this study. This scale includes 6 items meant to assess momentary anxiety (ie, “Right now I am tense”), and participants were prompted to indicate how much they agreed with the statements provided; responses could range from 1 (not at all) to 4 (very much so).

#### State Mindfulness and Self-Compassion

As we did for our prior feasibility study within this area, we developed a study-specific 5-item scale to assess mindfulness/self-compassion that leverages items from the Toronto Mindfulness Scale [33] and the State Self-Compassion Scale [34]. As we asked participants questions every 2 minutes, we used a shortened scale to reduce participant burden and to make sure participants did not have their attention drawn away from the intervention for too long.

For our study-specific scale, we selected 2 items from the Toronto Mindfulness Scale with high loadings on the 2 factor structure of the scale, and we also selected 3 questions from the Self Compassion Scale that captured essential qualities related to self-compassion (ie, lowered self-judgment). We summed all items on this scale, leaving us with an overall mindfulness/self-compassion score that could range from 3 to 23 (high mindfulness/self-compassion). Specifically, we used the 5 items outlined in Textbox 1.

**Items for study specific mindfulness/self-compassion scale.**

**State Self-Compassion Scale Questions** (scale 1-5; 1=not at all true for me, 5=very true for me):

- I’m giving myself the caring and tenderness I need
- I feel intolerant and impatient toward myself. (reverse coded)
- I’m keeping things in perspective

**Toronto Mindfulness Scale Questions** (scale 0-4; 0=not at all, 4=very much so):

- I was curious about what I might learn about myself by taking notice of how I react to certain thoughts, feelings or sensations
- I was more invested in just watching my experiences as they arose, than in figuring out what they could mean

#### Feasibility and Acceptability

We assess feasibility and acceptability within this study with seven study-specific quantitative questions: (1) How likely would you be to recommend this intervention to someone you care about from the Black community that is dealing with acute stress/anxiety? (Variable: “Likely to recommend;” 0: not at all likely; 50: moderately likely; 100: definitely likely). (2) To what extent was the intervention harmful or helpful for your stress/anxiety? (“Helpful for Anxiety;” 0: harmful; 50: no impact; 100: incredibly helpful). (3) To what extent did you find it difficult or easy to engage with the intervention? How practical and logistically easeful did it feel? (“Easy to Engage With;” 0: extremely difficult; 50: neutral; 100: as easy as I could imagine). (4) To what extent do you feel like the study changed your ability to be present with your thoughts and emotions? (“Improved Ability to be Present;” 0: much less able to be present; 50: no change; 100: much more able to be present). (5) To what extent do you think the study changed the way you feel about yourself? (“Improved Relationship with Self;” 0: much worse about myself; 50: no change; 100: much better about relationship to self). (6) To what extent do you feel that this intervention was made for people like you? (“Made for You;” 0: not at all; 50: somewhat; 100: exactly for me). (7) How helpful/harmful do you think it would be to experience and work with this intervention over a longer period of time (ie, daily for 1 to 2 weeks)? (“Longer Engagement Helpful;” 0: very harmful; 50: no difference; 100: very helpful).

Additionally, participants were asked open-ended qualitative questions about the number of days they feel they would need to engage with “healing attempt” for lasting benefit, as well as their likes, dislikes, and suggestions for improvement for the intervention.

### Analyses

#### Overview

We preregistered the analyses in this study [35]. All analyses were conducted in R (R Core Team) using the “Scan” package [36], as this software is designed for the analysis of various kinds of single-case experiments. In this study as well as our prior 2 studies of “healing attempt,” we analyzed all results using Tau-U analysis. This analytical approach entails evaluating the extent to which one’s data between 2 different study phases do not overlap (ie, the extent to which data in 1 phase is significantly higher or lower than data from another phase). Additionally, the core benefit of these analyses is that they allow one to control for data trends in one’s analyses, enabling us to account for any natural changes in our outcomes of interest (ie, anxiety and mindfulness/self-compassion) when analyzing our results. Each Tau-U model conducts 4 statistical tests: (1) A versus B (baseline vs intervention); (2) A versus B–trend A (baseline vs intervention, controlling for the trend in the baseline phase); (3) A versus B+trend B (baseline vs intervention, incorporating within intervention phase changes); and (4) A versus B+trend B–trend A (baseline vs intervention, controlling for baseline trends and incorporating within intervention phase changes).

Tau-U values typically range between –1 and +1, with the magnitude of the value serving as an estimate of the extent of the nonoverlap between one’s 2 study phases. Furthermore, a positive Tau-U value would indicate that “healing attempt” increased an outcome of interest, whereas a negative Tau-U value would indicate a decrease in an outcome of interest.

The main outcomes in this study are overall Tau-U models that are a composite of individual Tau-U models that are conducted for each participant; the “Scan” package combines these individual Tau-U models by weighting individual Tau-U values for each participant by their standard errors and then averaging values across participants. As we had 2 main outcomes in this study—state anxiety and mindfulness/self-compassion—we had 2 overall Tau-U models as our main outcomes.

#### Power

We used the Scan package to conduct a priori power analyses for this multiple baseline experiment. These analyses indicated that we would be sufficiently powered (80%) to detect a medium intervention effect (*d=*0.5) with α error rate <5% if we had at least 3 participants and at least 5 baseline measurement periods per participant. These analyses assumed a fixed rate of 12 intervention measurement periods per participant, based on the length of “healing attempt.” Thus, as our study featured 4 participants and at least 5 baseline measurements per participant, we were sufficiently powered in this study. We report the results of our power analyses in Table S1 in Multimedia Appendix 1; these power analyses were also used as the basis for the study design of our prior 2 feasibility studies of “healing attempt.”

## Results

### Overview

The demographics of the 4 participants in this study are presented in Table 1.

**Table 1:** Demographics of the 4 participants.

Participant	Gender	Age	Income (US $)	Education	Meditation familiarity	Music for support	Trait anxiety (STAIT-5)^a^	Racism as a cause of anxiety
Case 1	Female	33	25,000-34,999	Bachelor’s degree	Not at all (“what is mindfulness/meditation?”)	Rarely	18	60
Case 2	Trans man	25	<15,000	Bachelor’s degree	Somewhat (“I’ve meditated once or twice”)	All the time	15	60
Case 3	Female	22	<15,000	Bachelor’s degree	Somewhat (“I’ve meditated once or twice”)	All the time	20	70
Case 4	Female	26	<15,000	Bachelor’s degree	Somewhat (“I’ve meditated once or twice”)	All the time	12	80

^a^STAIT-5: State-Trait Anxiety Inventory-Trait-5.

**Figure 2:**
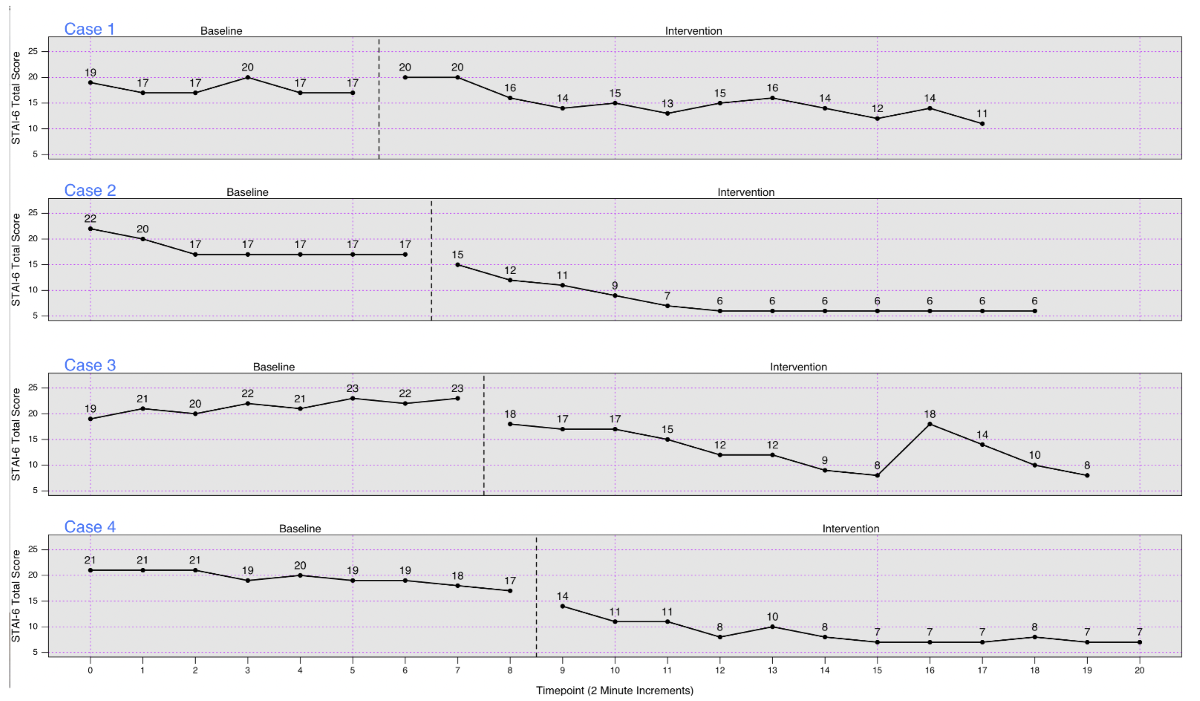
State anxiety scores for all participants. STAI-6: State Trait Anxiety Inventory-6.

**Figure 3:**
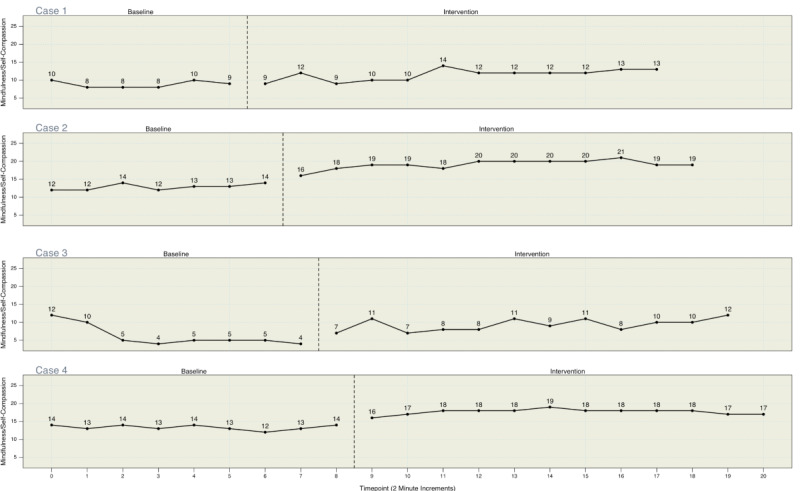
Mindfulness/self-compassion scores for all participants.

Figures 2 and 3 present the individual measurements of state anxiety and mindfulness/self-compassion, respectively, for each participant at each timepoint within the study. As presented in Figure 2, each participant showed elevated, stable anxiety scores during the baseline phase. Upon switching to the experimental/treatment phase, anxiety decreased for each participant. For cases 2-4, there was an immediate decrease in anxiety, which was lower at every assessment in the experimental phase than in each assessment in the baseline phase. For case 1, anxiety did not decrease for the first 2 assessments, but the subsequent 10 were all lower than any assessment point in the baseline phase. Figure 3 shows a similar pattern of results for increasing mindfulness/self-compassion upon entry into the experimental phase.

Additionally, Tables 2 and 3 present the results of the overall Tau-U models assessing the impact of the intervention on state anxiety and mindfulness/self-compassion, respectively. For our overall Tau-U model for state anxiety, all Tau-U values were negative and significant (*P*<.001), indicating that the intervention decreased anxiety even when one corrects for natural changes in anxiety that may have occurred during the study. For our overall Tau-U model assessing the impact of the intervention on mindfulness/self-compassion, all Tau-U values were positive and significant (*P*<.001), indicating that the intervention served to increase mindfulness/self-compassion.

**Table 2:** Overall Tau-U model assessing the impact of the intervention on state anxiety.

Model	Tau (95% CI)	SE	z	*P* value
A vs B	–0.93 (–1.20 to –0.66)	0.14	–6.76	<.001
A vs B–trend A	–0.88 (–1.20 to –0.55)	0.17	–5.28	<.001
A vs B+trend B	–0.80 (–0.99 to –0.61)	0.10	–8.40	<.001
A vs B+trend B–trend A	–0.66 (–0.82 to –0.50)	0.08	–8.05	<.001

**Table 3:** Overall Tau-U model assessing the impact of the intervention on mindfulness/self-compassion.

Model	Tau (95% CI)	SE	z	*P* value
A vs B	0.86 (0.59-1.13)	0.14	6.20	<.001
A vs B–trend A	0.88 (0.56-1.21)	0.17	5.34	<.001
A vs B+trend B	0.65 (0.46-0.84)	0.10	6.81	<.001
A vs B+trend B–trend A	0.57 (0.41-0.73)	0.08	6.94	<.001

### Feasibility Results

The results of our quantitative feasibility and acceptability assessments are presented in Table 4. Practically all feasibility and acceptability metrics received high ratings from participants, replicating the results from our first 2 feasibility studies. “Improved relationship with self” received moderate feasibility scores. Table 5 presents the number of days participants believe they would need to engage with the intervention for lasting benefit, as well as participant likes and dislikes/suggestions for improvement for the intervention. The “# of days for lasting benefit” ranged from 3 to 14 days. Next, 2 participants indicated that they enjoyed the music, an additional participant indicated that they enjoyed the overall experience, and a final participant indicated that they liked the positivity of the offerings included in the intervention. There were minimal suggestions for improvement for the intervention, although 1 participant indicated that the spoken word included in the intervention made them sad.

**Table 4:** Feasibility and acceptability scores.

Participant	Likely to recommend	Helpful for anxiety	Easy to engage with	Improved ability to be present with emotions	Improved relationship with self	Made for you	Longer engagement helpful
Case 1	80	70	70	70	50	60	80
Case 2	100	100	100	90	80	90	100
Case 3	85	80	75	70	65	100	90
Case 4	85	100	90	95	80	90	90
Mean	88	88	84	81	69	85	90

**Table 5:** Number of days for lasting benefit, likes and dislikes/suggestions for improvement.

Participants	# of days for lasting benefit	Likes	Dislikes/Suggestions for improvement
Case 1	3	The positivity of the offerings	None
Case 2	14	Overall experience	None
Case 3	4	Music; singing	None
Case 4	7	Music	Spoken word was sad

## Discussion

### Principal Findings

The goal of this study was to investigate the feasibility and preliminary efficacy of “healing attempt”—a brief, digital music–based mindfulness intervention for race-based anxiety in the Black community—in a sample of Black Americans with elevated race-based anxiety and little to no meditation experience. Consistent with our hypothesis, we found evidence that “healing attempt” decreased anxiety and increased mindfulness/self-compassion in our sample; additionally, the intervention received strong feasibility and acceptability ratings as well. These results are in line with our prior 2 feasibility studies of “healing attempt,” which also found the intervention to decrease state anxiety and increase mindfulness/self-compassion with high feasibility and acceptability [30,31]. Thus, this study adds to the evidence that “healing attempt” may be a feasible intervention for race-based anxiety in the Black community and inspires future research to more deeply investigate this possibility. Several aspects of this study warrant further comment.

### Potential Mediating Role of Mindfulness and Self-Compassion

In this study as well as in our prior work, we found a simultaneous increase in participants’ state mindfulness/self-compassion as well as a decrease in participants’ state anxiety. Therefore, it is plausible that heightened mindfulness and self-compassion could serve as mechanisms for reducing race-based anxiety. To be sure, this hypothesis was not formally tested here, though our findings are consistent with this possibility and set the stage for future tests of the mediating role of mindfulness/self-compassion. Mindfulness, with its emphasis on present-moment awareness, equips individuals to navigate anxious thoughts and emotions by anchoring them to their present-moment experience. Thus, mindfulness may disrupt the cyclical, anticipatory, and distressed states of mind that occur when one experiences heightened anxiety. Similarly, self-compassion is defined by self-kindness and nonjudgment, 2 properties of mind that may counteract the self-criticism that exacerbates anxiety. Our findings are consistent with a body of research that underscores the therapeutic potential of mindfulness and self-compassion in anxiety management [14,37-40]. The “healing attempt” intervention’s focus on nurturing these qualities may allow for a holistic and potentially powerful approach to alleviating race-based anxiety symptoms in Black Americans.

### High Accessibility and Ecological Validity

The digital nature of “healing attempt” marks a departure from traditional, in-person therapeutic approaches, and likely contributed to the results of our study. By enabling participants to engage in the intervention within the comfort of their own environments via Zoom, we have overcome several common barriers (eg, travel times and lack of availability/opportunity to engage in treatment) that can be associated with seeking psychological support in diverse populations [41,42]. The accessible nature of the intervention not only eliminates logistical challenges for our participants but also enhances the ecological validity of the study, as participants accessed the intervention in real-world contexts and often from the comfort of their homes. This novel approach has the potential to revolutionize existing approaches to mental health interventions, particularly for communities of color where barriers to access treatment are prominent.

### Cultural Relevance

The feasibility and potential efficacy of “healing attempt” is likely driven by its cultural relevance. This intervention’s design is centered around culturally relevant and communal healing, wherein participants partake in an intervention crafted by Black artists, made specifically for Black Americans. This intentionality may bolster the positive effects of the intervention. While most mindfulness studies have concentrated on predominantly White populations, our study has prioritized addressing the specific needs and challenges faced by Black Americans. Consistent with existing research [43-46], our findings suggest that the intervention’s alignment with participants’ cultural identities contributes to its feasibility for reducing race-based anxiety.

### Limitations

Despite the strengths of this study, it is not without its limitations. First, the current design includes a small sample of N=4 adults in a within-subjects design, 2 factors that may limit the generalizability of the study’s findings. Future research should examine “healing attempt” in a between-subjects randomized feasibility trial, which would warrant a greater sample size of at least 30 participants per group (per foundational studies on feasibility studies that recommend this sample size) [47-49].

Second, the authors of the “Scan” package note that the method used to calculate the CIs for the Tau-U models is based on a conservative approach, ultimately leading to relatively wider CIs around our Tau-U point estimates. Though wider CIs may indicate greater uncertainty for our Tau-U estimates, the significant findings achieved in our study actually reflect the robustness of the results, which were achieved under more stringent, conservative criteria.

Third, the mindfulness/self-compassion scale used in the study consisted of items selected from 2 full, validated scales (the Toronto Mindfulness Scale and Self-Compassion Scale). The decision to use a shorter scale was to reduce participant burden and increase intervention engagement; however, the lack of full scales for these constructs may have impacted the findings. Relatedly, due to this decision, in this study, we cannot parse out the relative changes of mindfulness versus self-compassion that may have been driven by the intervention. Using more fine-grained assessments of mindfulness and self-compassion in future studies can yield novel insights into how “healing attempt” impacts these 2 related constructs.

### Future Directions

#### “healing attempt” as a Treatment for Trait (ie, Persisting) Anxiety

Our findings in this study demonstrated that the intervention can reduce state (ie, momentary) anxiety. The subsequent phase in this body of research is confirming the viability of “healing attempt” as a treatment for chronic symptoms of anxiety (ie, trait anxiety); furthermore, forthcoming studies can also ascertain the potential of “healing attempt” to create lasting increases in mindfulness and self-compassion. A between-subjects randomized feasibility trial would offer the opportunity to explore these research questions. Moreover, this experimental design would enable us to investigate factors that moderate the effectiveness of the intervention as well.

#### Testing Intervention Effectiveness for Race-Based Anxiety in Other Minoritized Populations

Another next step in this line of research can be screening other diverse populations who experience race-based discrimination and testing the effect of the intervention on race-based anxiety among these groups. Although the music in “healing attempt” is rooted in Black American music traditions, the songs and meditations in the intervention are also general enough to potentially support anxiety reduction and well-being in other communities. Future feasibility studies can therefore assess this research domain.

#### Self-Administered Form of the Intervention

Examining self-administration of “healing attempt” is a significant next step, potentially offering increased convenience and fewer barriers to access the intervention (eg, stigma and logistical challenges). Deploying a self-administered form of the intervention would further take advantage of the key strengths of “healing attempt,” which include its scalability and digital format.

#### Testing Intervention Dissemination

Another next step in this line of inquiry is testing different routes of intervention delivery as such studies can allow us to further evaluate the impact of the intervention within real-life contexts. Excitingly, the digital nature of the intervention opens many possibilities for disseminating the intervention on the internet. For instance, prior pilot studies have demonstrated that the social media website Tumblr may represent a feasible pathway for implementing single-session interventions [50]. Accordingly, future studies can assess disseminating “healing attempt” via social media platforms as well. Additionally, as “healing attempt” is a music-based intervention, future studies can also explore music streaming platforms as a way to disseminate the intervention, as this approach would provide a cost-effective and accessible way for Black Americans to access the intervention. Implementation studies could subsequently assess the impact of the intervention on mental health in the Black community and additionally assess patterns of usage of the intervention, ultimately allowing us to update the intervention and our implementation approach to maximize impact and effectiveness.

### Conclusions

This investigation aimed to explore the feasibility of “healing attempt” as a treatment for race-based anxiety in Black Americans with little-to-no mindfulness experience. Replicating our previous results, we found that the intervention improved state anxiety as well as mindfulness/self-compassion and received strong feasibility and acceptability scores. Limitations of this study include the within-subjects nature of the intervention, the small sample size, and the focus on momentary versus lasting improvements in our health targets. Future investigation can assess whether the intervention can produce lasting changes in race-based anxiety, mindfulness, and self-compassion in a between-subjects randomized trial, test “healing attempt” for improving race-based health issues in other groups, test self-administered forms of the intervention, and assess different strategies for intervention dissemination. Overall, this study provides yet more evidence that “healing attempt” is a feasible intervention for improving well-being within the Black community, and inspires exciting future directions that can be explored in future work.

## Appendix

Multimedia Appendix 1 Power analysis for 5 baseline periods and 12 intervention periods for 3 participants to detect a moderate intervention effect (d=0.5) using overall Tau-U analyses. Conducted using 500 Monte Carlo simulations.

## Abbreviations

MBSR: mindfulness-based stress reduction
STAI-6: State Trait Anxiety Inventory-6
STAIT-5: State-Trait Anxiety Inventory-Trait-5
