# Supplementary material for: A Brief, Digital Music-Based Mindfulness Intervention for Black Americans With Elevated Race-Based Anxiety and Little-to-No Meditation Experience (“healing attempt"): Replication and Extension Study
Source: JMIR Form Res. 2023 Nov 24;7:e53268. doi: 10.2196/53268 (PMC10709790; doi:10.2196/53268)
Supplement: Multimedia Appendix 1 [file formative_v7i1e53268_app1.docx]

#### **Multimedia Appendix 1**

Power analysis for 5 baseline periods and 12 intervention periods for 3 participants to detect a moderate intervention effect (d = 0.5) using overall Tau U analyses. Conducted using 500 monte-carlo simulations.

| Method | Power | Alpha Error | Alpha:Beta | Correct | p-value |
| --- | --- | --- | --- | --- | --- |
| Overall Tau-U Analyses | 84% | 4% | 1:4.1 | 89.9 | 0 |
